# Dose Dependent Antimicrobial Cellular Cytotoxicity—Implications for *ex vivo* Diagnostics

**DOI:** 10.3389/fphar.2021.640012

**Published:** 2021-08-10

**Authors:** Ana Copaescu, Phuti Choshi, Sarah Pedretti, Effie Mouhtouris, Jonathan Peter, Jason A. Trubiano

**Affiliations:** ^1^ Centre for Antibiotic Allergy and Research, Department of Infectious Diseases, Austin Health, Heidelberg, VIC, Australia; ^2^ Allergy and Immunology Unit, University of Cape Town Lung Institute, Cape Town, South Africa; ^3^ Division of Allergy and Clinical Immunology, Department of Medicine, University of Cape Town, Cape Town, South Africa; ^4^ Department of Oncology, Sir Peter MacCallum Cancer Centre, The University of Melbourne, Parkville, VIC, Australia; ^5^ Department of Medicine (Austin Health), The University of Melbourne, Heidelberg, VIC, Australia; ^6^ The National Centre for Infections in Cancer, Peter MacCallum Cancer Centre, Melbourne, VIC, Australia

**Keywords:** delayed hypersensitivity reaction, drug allergy, severe cutaneous adverse reaction, T-cell, enzyme linked ImmunoSpot, cytotoxicity, flow cytometry, lactate dehydrogenase

## Abstract

**Introduction:***Ex vivo* and *in vitro* diagnostics, such as interferon-γ (IFN-γ) release enzyme linked ImmunoSpot (ELISpot) and flow cytometry, are increasingly employed in the research and diagnostic setting for severe T-cell mediated hypersensitivity. Despite an increasing use of IFN-γ release ELISpot for drug causality assessment and utilization of a range of antimicrobial concentrations *ex vivo*, data regarding antimicrobial-associated cellular cytotoxicity and implications for assay performance remain scarcely described in the literature. Using the measurement of lactate dehydrogenase (LDH) and the 7-AAD cell viability staining, we aimed via an exploratory study, to determine the maximal antimicrobial concentrations required to preserve cell viability for commonly implicated antimicrobials in severe T-cell mediated hypersensitivity.

**Method:** After an 18-h incubation of patient peripheral blood monocytes (PBMCs) and antimicrobials at varying drug concentrations, the cell cytotoxicity was measured in two ways. A colorimetric based assay that detects LDH activity and by flow cytometry using the 7-AAD cell viability staining. We used the PBMCs collected from three healthy control participants with no known history of adverse drug reaction and two patients with a rifampicin-associated drug reaction with eosinophilia and systemic symptoms (DRESS), confirmed on IFN-γ ELISpot assay. The PBMCs were stimulated for the investigated drugs at the previously published drug maximum concentration (*Cmax*), and concentrations 10- and 100-fold above.

**Results:** In a human immunodeficiency virus (HIV) negative and a positive rifampicin-associated DRESS with positive *ex vivo* IFN-γ ELISpot assay, use of 10- and 100-fold *Cmax* drug concentrations decreased spot forming units/million cells by 32–100%, and this corresponded to cell cytotoxicity of more than 40 and 20% using an LDH assay and 7-AAD cell viability staining, respectively. The other antimicrobials (ceftriaxone, flucloxacillin, piperacillin/tazobactam, and isoniazid) tested in healthy controls showed similar dose-dependent increased cytotoxicity using the LDH assay, but cytotoxicity remained lower than 40% for all *Cmax* and 10-fold *Cmax* drug concentrations except flucloxacillin. All 100-fold *Cmax* concentrations resulted in cell death >40% (median 57%), except for isoniazid. 7-AAD cell viability staining also confirmed an increase in lymphocyte death in PBMCs incubated with 10-fold and 100-fold above *Cmax* for ceftriaxone, and flucloxacillin; however, piperacillin/tazobactam and isoniazid indicated no differences in percentages of viable lymphocytes across concentrations tested.

**Conclusion:** The LDH cytotoxicity and 7-AAD cell viability staining techniques both demonstrate increased cell death corresponding to a loss in ELISpot sensitivity, with use of higher antimicrobial drug concentrations for *ex vivo* diagnostic IFN-γ ELISpot assays. For all the antimicrobials evaluated, the use of *Cmax* and 10-fold *Cmax* concentrations impacts cell viability and potentially affects ELISpot performance. These findings inform future approaches for *ex vivo* diagnostics such as IFN-γ release ELISpot.

## Introduction

T-cell mediated adverse drug reactions can be life-threatening, with mortality rates up to 50% in certain severe cutaneous adverse drug reactions (SCAR) (Peter et al., 2017). Antimicrobial drugs are responsible for a significant burden of these reactions; more so in high burden tuberculosis (TB) and human immunodeficiency virus (HIV) settings (Blumenthal et al., 2019). Delayed hypersensitivity reactions to antimicrobials frequently occur in the setting of multidrug regimens e.g., TB infection; and rapid, accurate, and safe diagnostic tools to aid the identification and exclusion of the offending drug are critical (Lehloenya et al., 2020). Drug provocation testing is often considered contraindicated in most settings of severe, life-threatening adverse drug reactions; while systemic reactions to *in vivo* diagnostics such as patch testing has been reported, especially in the context of HIV infection (Lehloenya et al., 2020). Thus, *in vitro* and *ex vivo* diagnostics are appealing and have been increasingly employed in the evaluation of presumed T-cell mediated hypersensitivity reactions (Lehloenya et al., 2020).

The Enzyme-Linked ImmunoSpot (ELISpot) assay, an *ex vivo* diagnostic, was initially introduced in 1983 (Sedgwick and Holt, 1983) and detects locally secreted cytokine molecules, such as IFN-γ, using antibody-coated plates offering both a qualitative but also a quantitative specific protein measurement, measuring cytokine production on a per-cell basis (Cox et al., 2006). Successful IFN-γ production requires adequate, live T lymphocytes. Despite an increasing use of IFN-γ release ELISpot for drug causality assessment and utilization of a range of antimicrobial concentrations *ex vivo*, data regarding antimicrobial-associated cellular cytotoxicity and implications for assay performance remain scarcely described in the literature.

## Materials and Methods

We used the peripheral blood monocytes (PBMCs) from three control participants and two patients with IFN-γ ELISpot confirmed rifampicin-associated drug reaction with eosinophilia and systemic symptoms (DRESS). To understand the required blood quantity for each assay, one needs to keep in mind that, for a healthy control, 9 ml of blood gives us approximately 1 × 10^7^ PBMC. About 3 × 10^7^ (or 27 ml of blood) is required for each executed assay (e.g., ELISpot, LDH, or flow cytometry).

For these patients, the blood was collected less than 2 weeks after their acute DRESS reaction and they were not known to have any other drug hypersensitivities. PBMCs were isolated from heparinized peripheral blood [ethylenediaminetetraacetic acid (EDTA) tubes] by density gradient centrifugation (using polysaccharide Ficoll Paque®) as described previously (Trubiano et al., 2018) and preserved in liquid nitrogen or a −80° freezer until assessment. After thawing, prior to sample use, cell viability was measured using 0.4% trypan blue staining solution (Thermo Fisher Scientific®) and the Countess® automated cell counter (Fisher Scientific, 2010).

### IFN-γ ELISpot Assay

The INF-γ ELISpot assay was performed using a negative (unstimulated, media alone) and a positive control (anti-human CD3 antibody, 1-D1K, Mabtech®). The drug concentrations used for ELISpot and corresponding to the *Cmax* and the 10-fold value are illustrated in Supplementary Table S1 **.** The average number of spots for the test and unstimulated wells were calculated. A positive response was defined as equal or greater than 50 spot forming units (SFU)/million cells after background (unstimulated control) removal as per previously published definitions (Keane et al., 2012; Keane et al., 2014). The method is illustrated in Supplementary Figure S1. Spots were analyzed automatically with an AID ELISpot Reader (software version 7.0) (Mabtech®).

### Drug Concentrations for Cytotoxicity Assays

The penicillin derivatives were provided by the local hospital pharmacies (Austin Health, Australia, and University of Cape Town Private Hospital, South Africa): ceftriaxone (50 mg/ml), flucloxacillin (50 mg/ml), and piperacillin-tazobactam (200 mg/ml). The remaining drugs were commercially bought products: isoniazid (10 mg/ml; Sigma®, I3377-50G) and rifampicin (60 mg/ml; Sigma®, R3501-250 MG). The cells (2 × 10^5^/well) were added in triplicates for the control patients and duplicates for the two DRESS patients to three pre-determined drug concentrations: including *Cmax*, 10-fold above and 100-fold above (Table 1) and then allowed to incubate for 18 h at 37°C with 5% CO_2._ The initial concentration tested for flucloxacillin and ceftriaxone was 200 μg/ml, 150 μg/ml for piperacillin-tazobactam and 25 μg/ml for isoniazid and rifampicin (Table 1).

**TABLE 1:** Drug concentrations used for LDH assay and flow cytometry using the 7-AAD viability stain on lymphocytes.

Drugs	Concentrations (μg/ml)		
	#1	#2	#3
Ceftriaxone	**200**	**2,000**	20,000
Flucloxacillin	**200**	**2,000**	20,000
Piperacillin–tazobactam	**150/18.75**	**1,500/187.5**	15,000/1,875
Isoniazid	**50**	**500**	5,000
Rifampicin	**25**	**250**	2,500

Note: The concentrations in bold characters are the ones currently used for the ELISpot assay.

### Cytotoxicity Based Lactate Dehydrogenase Assay

Cytotoxicity or cell death is measured with assays using markers of apoptosis and/or necrosis of target cells (Saade et al., 2012; Specian et al., 2016). The colorimetric measurement of lactate dehydrogenase (LDH), is a reliable and commonly used technique (Specian et al., 2016). LDH, the cytosolic enzyme present in nucleated cells, is released into the extracellular media once the cell membrane is disrupted. The LDH activity is measured indirectly by the transformation of lactate in pyruvate with the reduction of nicotinamide adenine dinucleotide (NAD^+^) to NAD^+^H^+^ lactate (Specian et al., 2016; Abcam, 2019). The oxidation of the NADH^+^ that causes an increase in absorbance will be proportional to the LDH activity and therefore proportional to the number of lysed cells (Saade et al., 2012).

The LDH assay was performed according to the manufacturer’s suggested protocol (Abcam, 2019). H-Cytotox, 2019 (LDH Assay, Abcam® kit: ab65393). After addition of LDH reaction mix, an optimal 30 min incubation was used. The optical density of the plate was read with a FLUOstar Optima plate reader set at 450 nm with a 620 nm reference wavelength using the Optima version 2.1 software (BMG Labtech®). The percentage of cytotoxicity was calculated as per equation in Supplementary Figure S2. GraphPad Prism version 8.0 software was used for data analysis (GraphPad Software, La Jolla California United States, www.graphpad.com).

### Flow Cytometry Using the 7-AAD Cell Viability Staining

Lymphocyte viability (Saade et al., 2012) was assessed with the use of flow cytometry using the fluorescent DNA intercalator 7-AAD (7-aminoactinomycin). 7-AAD is a fluorescent derivative of actinomycin D that selectively binds to GC regions of the DN, exposed during cell death; and is frequently used to stain and exclude dead cells in flow cytometry (Zembruski et al., 2012).

Cryopreserved PBMCs were thawed and rested in R10 media at 37°C for 3 h. Cells (2−3 x 10^5^) were incubated for 18 h with antimicrobials tested. After incubation, cells were stained with CD3 Alexa Fluor 700 and CD4 PerCp Cy5.5 (BD Pharmingen) and CD8-APC AF750 (Invitrogen) for 30 min at 4°C, then washed with FACS buffer (PBS containing 1% bovine serum albumin and 0.05% sodium azide). After washing, PBMCs were stained with 7-AAD (BD Pharmingen) for 15 min. Cell events were acquired on a LSRII flow cytometer and FlowJo software was used to analyse FCS files. The gating strategy is detailed in Supplementary Figure S3.

This study was approved by both the Austin Health ethics committee and the Human Research Ethics committee of the University of Cape Town. The investigators obtained written informed consent from the participants.

We sought to determine the maximal antimicrobial concentrations for cell viability using the cytotoxicity based LDH assay and the 7-AAD cell viability staining for commonly implicated antimicrobials in severe T-cell mediated hypersensitivity—ceftriaxone, flucloxacillin, piperacillin-tazobactam, isoniazid, and rifampicin.

## Results

### Effects of Increasing Cytotoxicity on ELISpot Performance in Rifampicin DRESS Patient

**FIGURE 1:**
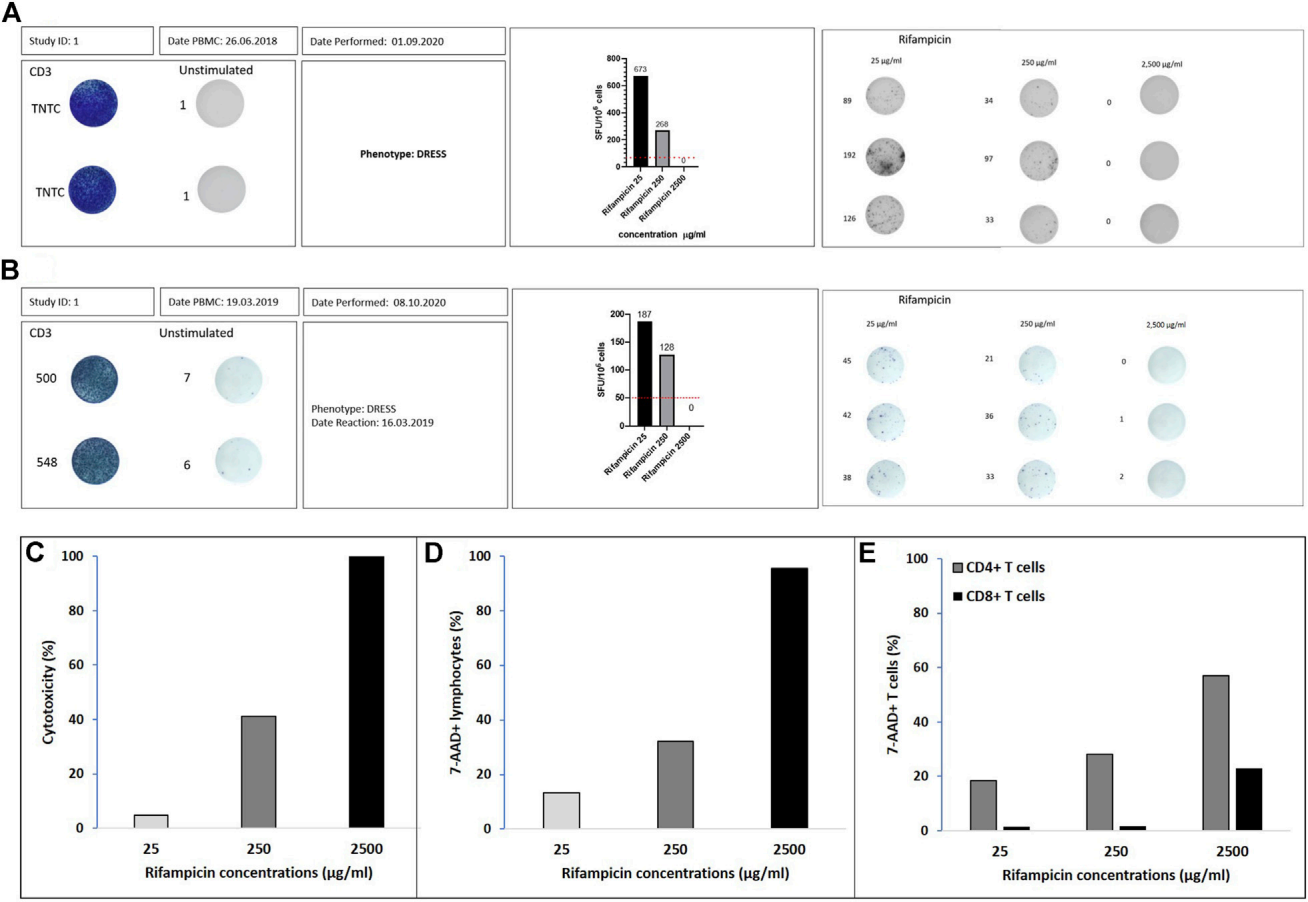
Rifampicin DRESS patients. IFN-γ ELISpot graphical illustration of **(A)** Australian patient and **(B)** south African patient. Positivity is defined by ≥ 50 spot forming unit (SFU)/million cells after background (unstimulated control) removal (dotted line on the corner right figures). Positive controls were done using CD3 and the unstimulated wells represent media alone. The stimulations with the different drug concentrations (µg/ml) were done in triplicates. The numbers adjacent to the wells represent the number of spots measured with the ELISpot reader. ELISpot graphical illustration for Rifampicin 25, 250, and 2500 μg/ml. Percentage of cytotoxicity assessed by **(C)** LDH assay, **(D)** flow cytometry with 7-AAD viability staining in lymphocytes and **(E)** flow cytometry with 7-AAD viability staining in CD4^+^ and CD8^+^ T cells.

Figures 1A,B show the SFU/million cells for the two DRESS patients with rifampicin as the offending drug (one HIV negative Figure 1A and one HIV co-infected Figure 1B). The SFU/million cells are highest at the lowest rifampicin concentrations (*Cmax* = 25 mg/ml, SFU/million cells of 673 and 187) and decrease in both patients at the 10-fold drug concentration to 268 (60% reduction) and 128 (32% reduction) respectively. At drug concentrations of 100-fold *Cmax* there are no T cell making IFN-γ and thus no SFU/million (100% reduction). Figures 1C,D show how cytotoxicity, measured by either the LDH assay or 7-AAD staining, increases with the 10- and 100-fold *Cmax* drug concentrations. In both the HIV negative and positive rifampicin-associated DRESS patients there was dose-dependent cytotoxicity with increasing concentrations of rifampicin. CD4 positive as well as CD8 positive lymphocytes showed the same pattern as illustrated in Figure 1E.

### Cytotoxicity Based LDH Assay

The number of viable cells initially added to the assay was 2 × 10^5^ per well, with cell recovery post thawing measured for all controls as follows: C1, 91%; C2, 76%, and C3, 80 and 75% for the patients that had a DRESS reaction to rifampin. The T cell activation in response to the offending drug for the patients was confirmed by a positive ELISpot assay as per previous definition (Figures 1A,B).

**FIGURE 2:**
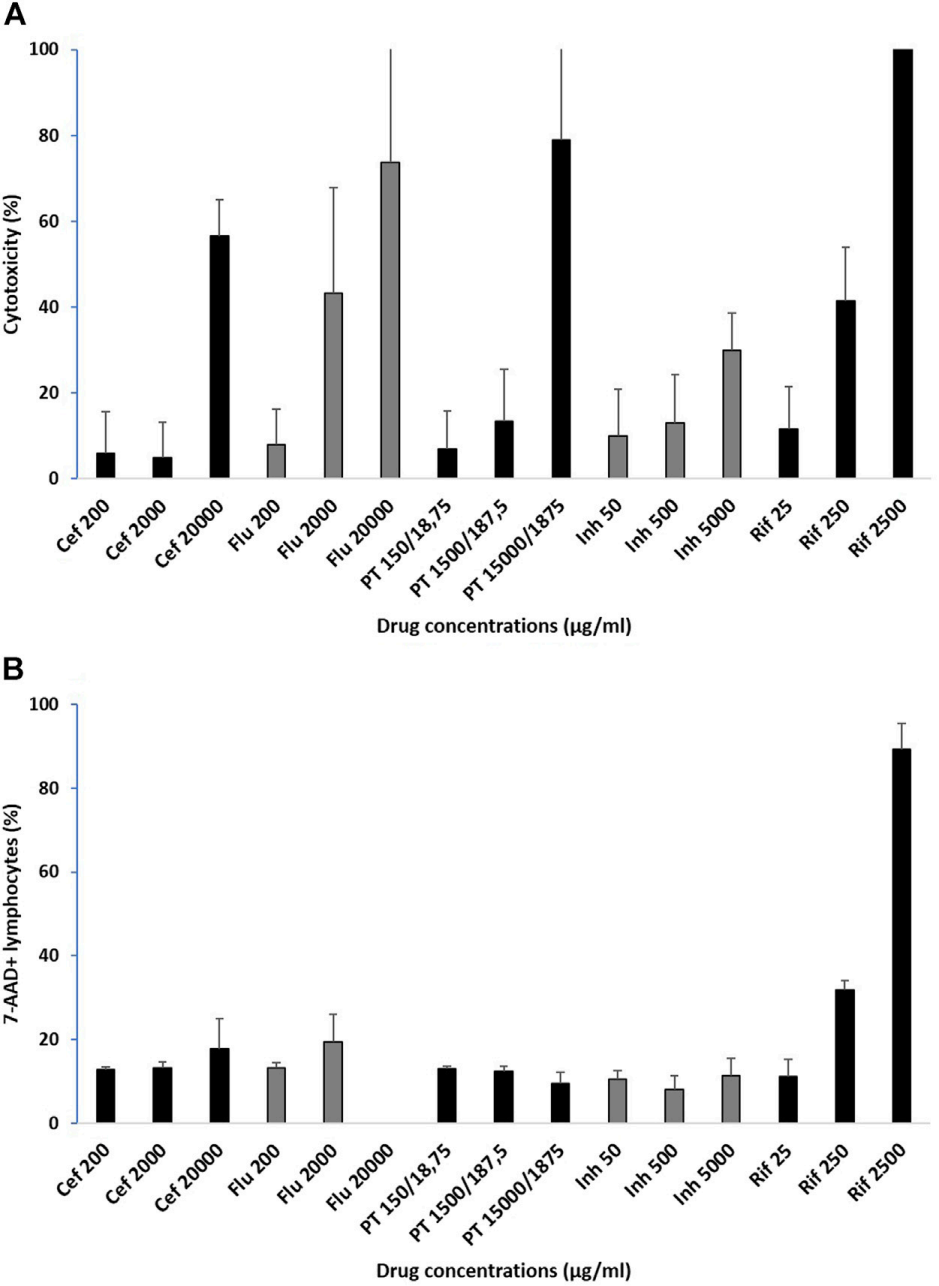
Percentage of cytotoxicity in healthy controls assessed by **(A)** LDH assay and **(B)** flow cytometry with 7-AAD viability staining in lymphocytes. Cytotoxicity average for 3 normal controls for the drugs included in the study at 3 pre-determined drug concentrations: *Cmax*, concentrations 10-fold and 100-fold, mean ± sd, Cef: ceftriaxone; Flu: flucloxacillin; PT: piperacillin-tazobactam; Inh: isoniazid; Rif: rifampicin.

The percentage of cytotoxicity seen in the LDH assay for all antimicrobials tested increased in a dose-dependent manner with increasing concentrations, but the extent of cytotoxicity varying by drug. The average cytotoxicity results for the three normal controls are illustrated in Figure 2A. The lowest concentrations, corresponding to previously published *Cmax* values and also used in *ex vivo* methods for ELISpots, showed a mean value of 8.4% (SD 8.4) cellular death. The mean percentage of cell death at 10-fold above *Cmax* was 23.1% (SD 20.8), but only rifampicin and flucloxacillin showed cytotoxicity more than 40%. In contrast, at the 100-fold *Cmax* drug concentrations the mean cell death was 97.7% (SD 113.1), and only isoniazid had a cytotoxicity less than 40% at this supra-physiological concentration.

### Flow Cytometry Using the 7-AAD Cell Viability Staining

The number of cells added to the assay was 3 × 10^5^ per well. The immunological intolerance for the patient with rifampicin DRESS was confirmed by a positive ELISpot assay (Figure 1B).

In control participants, lymphocyte cytotoxicity assessed by flow cytometry using 7-AAD viability staining showed variations with increasing concentrations, but results were drug-dependent in this assay. The average cytotoxicity results for the three normal controls are illustrated in Figure 2B. The lowest concentrations, corresponding to previously published *Cmax* values and also used in *ex vivo* methods for ELISpots, showed a mean 7-AAD staining of 11.9% (SD 2.4). For the 10-fold above *Cmax*, the mean was 17.3% (SD 8.7), but only rifampicin had staining above 20% at this drug concentration. For the 100-fold *Cmax* the mean 7-AAD staining was 31.9% (SD 35.1), with rifampicin very high. There was an increase in 7-AAD staining for PBMCs incubated with increasing concentrations of ceftriaxone, flucloxacillin (except the highest concentration which was not run due to technical difficulties) and rifampicin, no change with isoniazid and a slight decrease with piperacillin-tazobactam.

## Discussion

As IFN-γ release ELISpot is increasingly used for drug causality assessment in patients with life-threatening, severe immune-mediated adverse drug reactions (IM-ADRs), ELISpot has been shown to be an efficient *ex vivo* tool in the diagnosis of antibiotic-associated SCAR patients (Trubiano et al., 2018) by offering the benefit of observing the patient’s reaction to a drug without re-exposing the patient. To maximise assay sensitivity, it is advantageous to use the highest possible drug concentrations, especially in situations where the mechanism of drug-immune interaction is poorly defined. In this project, we examine antimicrobial-associated cellular cytotoxicity for common offending antimicrobials and its impact on assay performance. We describe two patients with rifampicin-associated DRESS (one HIV negative and one positive); as well as three control participants where the cytotoxicity of different antimicrobials used in clinical diagnostic was evaluated. Our main findings were: i) that in both the HIV negative and positive rifampicin-associated DRESS patients, there was a dose-dependent reduction in SFU/million cells in the ELISpots corresponding to increasing rifampicin-induced drug cytotoxicity; ii) for all the antimicrobials evaluated, the use of C*max* and 10-fold C*max* concentrations did not reach cytotoxicity seen with rifampicin and are therefore unlikely to affect ELISpot performance; and iii) for drugs such as piperacillin/tazobactam and isoniazid even concentrations as high as 100-fold C*max* did not result in cytotoxicity likely to reduce ELISpot performance.

ELISpot is an assay that requires optimization according to the population and drug of interest as well as the specific cytokine measured (Ji and Forsthuber, 2016). At high concentrations, drugs become toxic for cells *via* different mechanisms like overproduction of nitric oxide; generation of reactive oxygen species, and subsequent oxidative stress; mitochondrial dysfunction; and DNA damage. Not all drugs have equivalent cytotoxicity at the same concentrations and thus these experiments inform the optimization of diagnostic ELISpot for delayed IM-ADRs. Indeed, rifampicin appeared the most toxic of all tested drugs as illustrated with over 90% cytotoxicity at 100-fold C*max* concentrations and complete abrogation of SFUs in the ELISpot assay. Mechanisms of increased cytotoxicity with rifampicin are uncertain but the formation of drug-antibody complexes, which binds to platelet membrane activating complement and increasing cell death has been proposed (Combrink et al., 2020).

Various drug concentrations are described in the literature for *ex vivo* assays with varying units of measure including, millimoles (isoniazid, 1–4 mM; rifampicin, 0.1 mM) for tuberculosis related drugs (Usui et al., 2017) or micromoles (El-Ghaiesh et al., 2012) and mg/ml (Rozieres et al., 2009; Tanvarasethee et al., 2013; Sedlackova et al., 2018) for beta-lactam drugs. Thus, a strength of this project is the use of the measured *Cmax* as a reference to ensure that these units are comparable across drugs and concentrations are optimised as being the point of maximal bacterial cytotoxicity with minimum tolerable cellular injury (Supplementary Table S1).

We noted higher percentage of cytotoxicity with the LDH assay compared with the 7-AAD staining method. The 7-AAD staining involves a nuclear dye, while the LDH assay measures the oxidation of the NADH^+^ after cell membrane damage and LDH release into the extracellular medium. This distinction in assays could explain the difference observed in Figure 1. For the 7-AAD staining, all the drugs tested at the three concentrations, except rifampicin, showed relatively low cytotoxicity (<30%) while for the LDH assay, most drug concentrations 10-fold higher than *Cmax* resulted notable increases in cytotoxicity (>40%). The localisation of the damage—cytosolic (LDH assay) versus nuclear (7-AAD) may explain these differences, but the correlation of both assay with decreased SFUs in the rifampicin ELISpot provides clear data linking the differing degrees of cytotoxicity with decreasing assay performance.

Using flow cytometry for evaluating cytotoxicity can be performed with the uptake of labeled annexin V, propidium iodide or the uptake of fluorescent DNA probes 7-amino-actinomycin (7-AAD), as in our study (Saade et al., 2012). In apoptotic cells, membrane phosphatidylserine, which is located in the inner part of the membrane in healthy cells, translocates to the outer leaflet and is exposed to the external environment (Saade et al., 2012), where it binds with annexin V staining providing evidence of early cell death. Further, using 7-AAD dye gives information on loss of membrane integrity and later stages of cell death.

The study has limitations that include the inability to evaluate all the different drug concentrations for all antimicrobials in patients where the drug is known to be offending, and to directly match cytotoxicity data with ELISpot performance. However, the rifampicin data shows proof of concept and provides guidelines on when cytotoxicity levels are likely to effect assay performance. In addition, as with any laboratory assay, technical limits particularly for long-lasting cell cultures and complex manipulations as well as operator-dependent variability could influence results. Other potential considerations are sample irregularities in terms of time and condition of sample storage as well as drug formulation and preparation.

## Conclusion

Our results show that the known C*max* for each antimicrobial and the 10-fold increased drug concentrations showed a mean percentage of cytotoxicity below 30%, allowing a significant live population to evaluate in *ex vivo* experiments when these concentrations are employed. An increase in cytotoxicity was translated by fewer SFU in the ELISpot illustrating the impact of cell death on reduced ELISpot performance. These exploratory findings will inform approaches for *ex vivo* diagnostics such as IFN-γ release ELISpot increasingly utilized in drug causality assessments of rare severe T-cell mediated hypersensitivity.

## Data Availability

The original contributions presented in the study are included in the article/Supplementary Material, further inquiries can be directed to the corresponding author.

## Abbreviations

C*max*, maximum concentration recorded; CTL, cytotoxic T-cells; DRESS, drug reaction with eosinophilia and systemic symptoms; EDTA, ethylenediaminetetraacetic acid; ELISpot, enzyme linked immunospot; FBS, fetal bovine serum; IFN-γ, interferon-γ; LDH, lactate dehydrogenase; PBMCs, peripheral blood mononuclear cells; SCARs, severe cutaneous adverse reactions.
