# Corrigendum: Dose Dependent Antimicrobial Cellular Cytotoxicity—Implications for *ex vivo* Diagnostics

**DOI:** 10.3389/fphar.2021.758192

**Published:** 2021-09-02

**Authors:** Ana Copaescu, Phuti Choshi, Sarah Pedretti, Effie Mouhtouris, Jonathan Peter, Jason A. Trubiano

**Affiliations:** ^1^ Centre for Antibiotic Allergy and Research, Department of Infectious Diseases, Austin Health, Heidelberg, VIC, Australia; ^2^ Division of Allergy and Clinical Immunology, Department of Medicine, University of Cape Town, Cape Town, South Africa; ^3^ Allergy and Immunology Unit, University of Cape Town Lung Institute, Cape Town, South Africa; ^4^ Department of Oncology, Sir Peter MacCallum Cancer Centre, The University of Melbourne, Parkville, VIC, Australia; ^5^ Department of Medicine (Austin Health), The University of Melbourne, Heidelberg, VIC, Australia; ^6^ The National Centre for Infections in Cancer, Peter MacCallum Cancer Centre, Melbourne, VIC, Australia

**Keywords:** delayed hypersensitivity reaction, drug allergy, severe cutaneous adverse reaction, t-cell, enzyme link immunospot, cytotoxicity, flow cytoametry, lactate dehydrogenase

In the original article, we mistakenly omitted a statement from the Funding section. The statement reads as follows: “JP receives financial support from National Institutes of Health, award K43TW011178-02; the EDCTP2 programme supported by the European Union, grant number TMA2017SF-1981 and the South African National Research Foundation (NRF). PC receives a PhD fellowship from the Fogarty HATTP program, funded by the National Institutes of Health, award D43 TW010559-01”.

The authors apologize for this error and state that this does not change the scientific conclusions of the article in any way. The original article has been updated.

