# Supplementary material for: Dose Dependent Antimicrobial Cellular Cytotoxicity—Implications for ex vivo Diagnostics
Source: Front Pharmacol. 2021 Aug 10;12:640012. doi: 10.3389/fphar.2021.640012 (PMC8383281; doi:10.3389/fphar.2021.640012)

Supplementary Material

Dose Dependent Antimicrobial Cellular Cytotoxicity – Implications for *ex vivo* diagnostics

***Ana Copaescu^1^, *Phuti Choshi^2,^ Sarah Pedretti^2^, Effie Mouhtouris^1^, **Jonny Peter^2,3^, **Jason A Trubiano^1,4,5,6^**

*Co-first authors

**Co-Senior authors

^1^Centre for Antibiotic Allergy and Research, Department of Infectious Diseases, Austin Health, Heidelberg, Victoria, Australia

^2^Allergy and Immunology Unit, University of Cape Town Lung Institute, South Africa

^3^Division of Allergy and Clinical Immunology, Department of Medicine, University of Cape Town, South Africa

^4^Department of Oncology, Sir Peter MacCallum Cancer Centre, The University of Melbourne, Parkville, Victoria, Australia

^5^Department of Medicine (Austin Health), The University of Melbourne, Heidelberg, Australia

^6^The National Centre for Infections in Cancer, Peter MacCallum Cancer Centre, Melbourne, Victoria, Australia

**Supplement Table 1: Antimicrobial Concentration and Solubility Agent Summary**

| **Antimicrobial** | **Form/product^♦^** | **Diluent** | **Soluble concentration  (mg/ml)** | **Preferred testing (μg/ml)  concentrations** | ***Cmax* [21]***  **(μg/ml)** |
| --- | --- | --- | --- | --- | --- |
| **Penicillins and related drugs** | | | | | |
| Benzylpenicillin  Penicillin G | 1.2 g vial | water | 100 mg/ml | 200 & 2,000 | 12 (IM)  400 (IV) |
| Ampicillin and Amoxicillin | 1 g vials | water | 10 mg/ml | 200 & 2,000 | 83-112 (IV)  8-10 (PO) |
| Flucloxacillin | 1 g vial | water | 50 mg/ml  100mg/ml | 200, 2000 | 8.8-14.5(PO)  75.5-193.7 (IV) |
| Dicloxacillin | Capsule (crushed) | water | 10mg/ml | 20 & 200 | 4.2-193 (IV) |
| Piperacillin | Sigma powder | water | 10 mg/ml | 200 & 2,000 | 4.9-30.2 (IM)  70.7-451.8 (IV) |
| Piperacillin-tazobactam | 4/0.5g vial | water | 60 mg/ml  200 mg/ml | 18.75/150  187.5/1,500 | 259-300 (Pip)  27.9-34 (Taz) |
| Clavulanic acid | Sigma powder | water | 5 mg/ml | 10 & 100 | 2-3.5 (PO) |
| Amoxicillin clavulanate | 1.2 g vials | water | 10 mg/ml | 10 & 100 | 12.0/5.5 (PO) |
| **Cephalosporins and related drugs** | | | | | |
| Cefazolin | 1 g vial | water | 50 mg/ml | 200 & 2,000 | 34 (IM) |
| Cephalexin/ Cefalexin | Sigma powder | NH4OH | 20-50 mg/ml | 20 & 200 | 9-18 (PO) |
| Ceftriaxone | 1 g vial | water | 20 mg/ml  100 mg/ml | 200 & 2,000 | 123.2 (IV)  79.2 (IM) |
| Ceftazidime | 1 g vial | water | 50 mg/ml | 50 & 500 | 107-181 (IV) |
| Cefepime | 1 g vial | water | 50 mg/ml | 200 & 2,000 | 16.3-133 (IV) |
| **Carbapenems** | | | | | |
| Meropenem | 1 g vial | water | 50 mg/ml | 200 & 2,000 | 21.1-61.6 (IV) |
| Ertapenem | 1 g vial | water | 50 mg/ml | 100 & 1,000 | 103.3 (IV) |
| **Glycopeptides and Lipopeptides** | | | | | |
| Vancomycin | 1 g vials | water | 50 mg/ml | 50 & 500 | 13-40 (IV) |
| Teicoplanin | 400 mg vial | water | 50 mg/ml | 50 & 500 | 53.5-111.8 (IV)  7.1 (IM) |
| Telavancin | 250 or 750 mg vial | water | 15mg/ml | 50 & 500 | 96.7-232 (IV) |
| Dalbavancin | Sigma powder | Water | 2mg/ml | 50 & 500 | 133-423 (IV) |
| **Macrolides and Ketolides** | | | | | |
| Erythromycin | 250 mg tablets | DMSO | 25 mg/ml | 10 & 100 | 0.3-3.5 (PO) |
| Clarithromycin | 250 mg tablets | DMSO | 50 mg/ml | 2.5 & 25 | 2-4 (PO) |
| **Antifolate agents and other Synthetic Antibacterials** | | | | | |
| Sulfamethoxazole (Sulfonamide) | Sigma powder | DMSO | 50 mg/ml | 50 & 500 | 45-372 (PO) |
| 4-NitroSMX  [SMX-NO] | Sigma powder  Santa Cruz powder | DMSO | 100 mg/ml  3 mg/ml | 10 & 100 | 1-10 |
| Trimethoprim [TMP] | Sigma powder | DMSO | 50 mg/ml | 5 & 50 | 1.6-13.6 (PO) |
| Trimethroprim-Sulfamethoxazole [TMP/SMX] | 5 mg ampule (80[TMP]-400[SMX]) | not required (pre-diluted) | 16 mg [TMP]  80 mg [TMP]/ml | 50/250 | see TMP and SMX |
| **Nitroimidazole** | | | | | |
| Metronidazole | Sigma powder | water | 5 mg/ml | 25 & 250 | 5.1-40 (PO)  9.4-20 (IV) |
| **Quinolones and Fluroquinolones** | | | | | |
| Ciprofloxacin | 400 mg/200 ml IV bags | not required (pre-diluted) | 20 mg/ml | 10 & 100 | 3.5-4 (PO)  4 (IV) |
| Moxifloxacin | 400 mg/250 ml IV bags | not required (pre-diluted) | 1.6 mg/ml | 2 & 20 | 3.1-4.1 (PO) |
| **Other Antimicrobials** | | | | | |
| Linezolid | 600mg/300 ml IV bag | not required (pre-diluted) | 2mg/ml | 20 & 200 | 15.1 (IV)  11-21.1 (PO) |
| Doxycycline | capsules |  |  | 10 & 100 | 2.3-6.3 (PO) |
| Clindamycin | 300 mg vial | water | 50 mg/ml | 10 & 100 | 2.5-4.5 (PO)  4.8-16.8 (IV) |
| Fusidic acid | ACROS powder | water | 10mg/ml | 10 & 100 | 27 (PO)  52 (IV) |
| **Anti-Tuberculosis Drugs** | | | | | |
| Isoniazid | 100 mg tablets  Sigma powder | water | 40 mg/ml | 2.5 & 25 | 2.5-4.5 (PO) |
| Acetyl isoniazid | Santa Cruz powder | methanol | 10 mg/ml | 1, 10 | 0.5-6 (PO) |
| Ethambutol | 400 mg tablets  Sigma powder | water | 50 mg/ml | 2.5 & 25 | 5-10 (PO) |
| Pyrazinamide | 500 mg tablets  Sigma powder | DMSO | 50 mg/ml | 50 & 500 | 28.8-49.6 (PO) |
| Pyrazinoic acid | Clearsynth powder | water/ DMSO/methanol | 3 mg/ml | 1, 10 | 3-4.5 (PO) |
| Rifampicin | 600 mg vial  Sigma powder | Water  methanol | 60 mg/ml  50 mg/ml | 2.5 & 25 | 2-30 (PO) |
| 25-desacetyl Rifampicin | Santa Cruz powder | methanol | 1 mg/ml | 1, 10 | 1-11 (PO) |
| **Antifungal drugs** | | | | | |
| Fluconazole | 200 /100ml IV bag | not required (pre-diluted) | 2 mg/ml | 10 & 100 | 1.5-4.7 (IV)  1.55 (PO) |
| **Other Non-Antimicrobial Drugs** [22] | | | | | |
| Colchicine | Sigma powder | Ethanol | 50 mg/ml | 1 & 10 | 1.7-2.6 (PO) |
| Ibuprofen | Sigma powder | DMSO | 100 mg/ml | 5 & 50 | 39.2-76.6 (IV) |
| Meloxicam | Sapphire biosciences | DMSO | 20mg/ml | 2 & 20 | 0.9-1.81 (PO)  1.1-3.5 (IM) |
| Allopurinol | Sigma powder | NaOH | 10mg/ml | 5 & 50 | 3 (PO) |
| Oxipurinol | Sigma powder | DMSO | 50 mg/ml | 5 & 50 | 6.5 (PO) |
| Sulfasalazine | 500 mg tablets | NH_4_OH | 100 mg/ml | 5 & 50 | 50 (PO) |
| Lamotrigine | Sigma powder | DMSO | 10mg/ml | 5 & 50 | 0.58-4.63 (PO)  0.54-1.43 (IR) |

**Abbreviations:** DMSO, dimethyl sulfoxide; IM, intramuscular; IR, intra-rectal; IV, intravenous; NaOH, sodium hydroxide; NH_4_OH, ammonium Hydroxide; Pip, piperacillin; PO, per os/ by mouth; SMX, sulfamethoxazole; Taz, tazobactam; TMP, Trimethroprim

**♦Note:** Preferred in all situations is the powder product either from manufacturer (e.g. Sigma) or ‘powder for reconstitution’ from clinical vials. Tablets should be used only if all other means have been explored. If solubility is poor in H20 then explore alternative diluents rather than utilizing non-dissolved product.

*** *Cmax*** (pharmacology): *Cmax* is the maximum (or peak) serum concentration that a drug achieves in a specified compartment or test area of the body after the drug has been administered and before the administration of a second dose. It is a standard measurement in pharmacokinetics.

Reference values presented are for healthy adults. For every drug presented, the *Cmax* values were all extracted from the cited reference while recognizing the limit in terms on varied study methodology, doses used and administration routes for pharmacokinetics calculations.

**Supplement Figure 1**: **Detection of cytokine secretion using ELISpot**

Cytokine-specific coating antibody is added and incubated overnight at 4°C. The plate is washed with phosphate-buffered saline and PBMCs and drug(s) are added and incubated for 18-20 hours at 37°C. The following day, after adequate plate washing, biotin-conjugated detection antibody is added and incubated for 2 hours at room temperature followed by Streptavidin-bound enzyme that is subsequently incubated for 1 hour at room temperature. After the last wash, substrate is added (BCIP-NBT or TMB). Spot development is monitored for ~15 minutes (in dark). The plate is washed and left to dry overnight for a final reading on the 4^th^ day.

Each experiment is performed in triplicate with the patient’s PBMCs stimulated with the relevant antibiotic concentrations and in duplicate for healthy control (penicillin-exposed nonallergic).

**Abbreviations:** BCIP-NBT, 5-bromo-4-chloro-3-indolyl phosphate (BCIP)/nitro blue tetrazolium (NBT); PBMCs, peripheral blood mononuclear cells; TMB, tetramethyl benzidine.
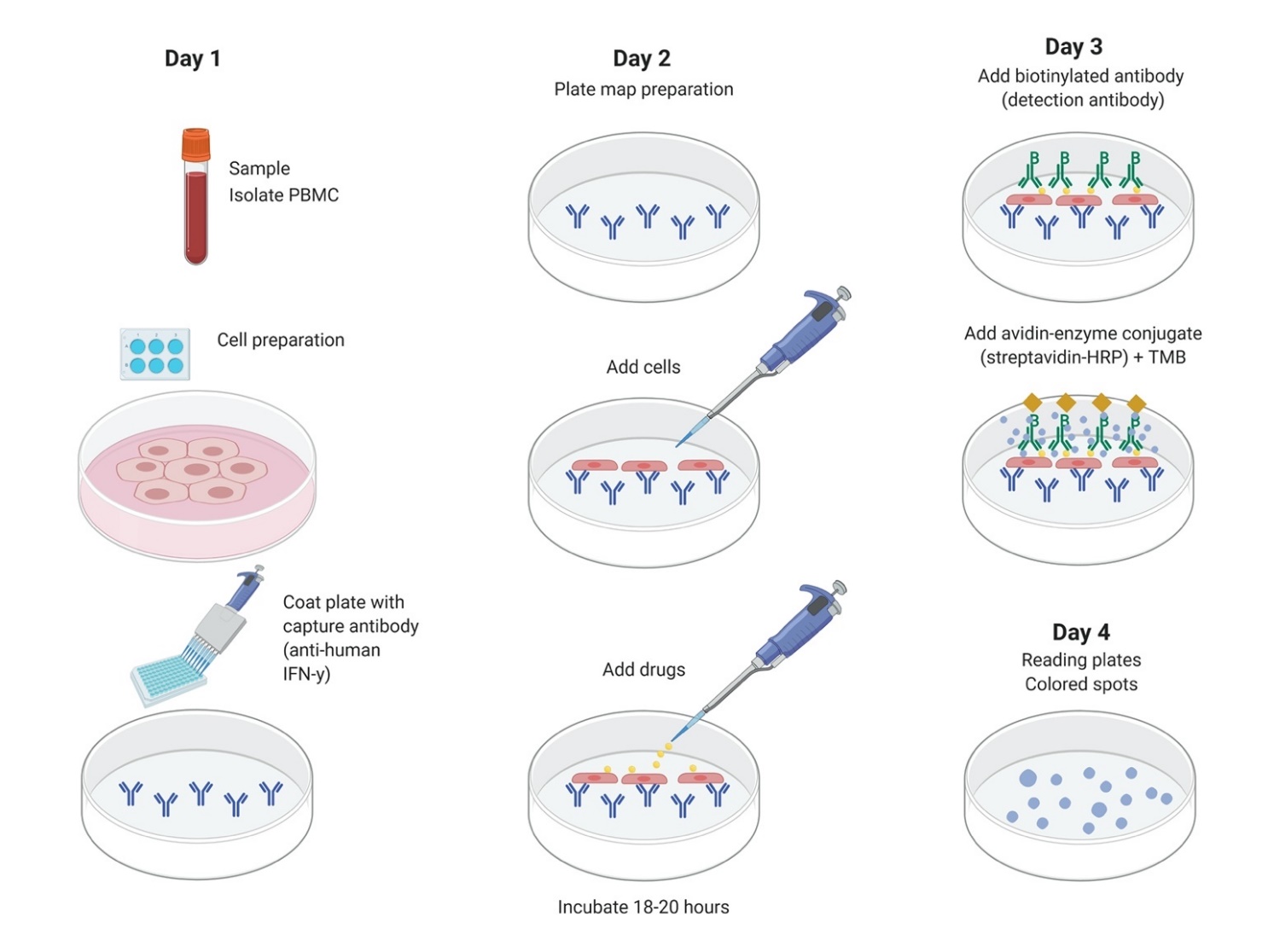


**Supplement Figure 2**: Cytotoxicity calculation equation

$$\boldsymbol{Cytotoxicity}\left( \boldsymbol{\%} \right)\boldsymbol{=}\left( \frac{\boldsymbol{(Test sample-Low control)}}{\boldsymbol{(High control-Low control)}}\boldsymbol{x 100} \right)$$

**Supplement Figure 3: Gating strategy for flow cytometry analysis.**

The gating strategy consisted of first A) gating lymphocytes based on FSC and SSC, then B) gating single cells based on FSC-A and FSC-H and finally C) gating 7-AAD positive cells as dead lymphocytes (7-AAD-PE-Cy5-A^+^ cells). For the rifampicin induced DRESS patient, in addition to the previous strategy D) a gating for CD3 positive T cells was added as well as E) a gating for CD4 positive and CD8 positive dead lymphocytes.


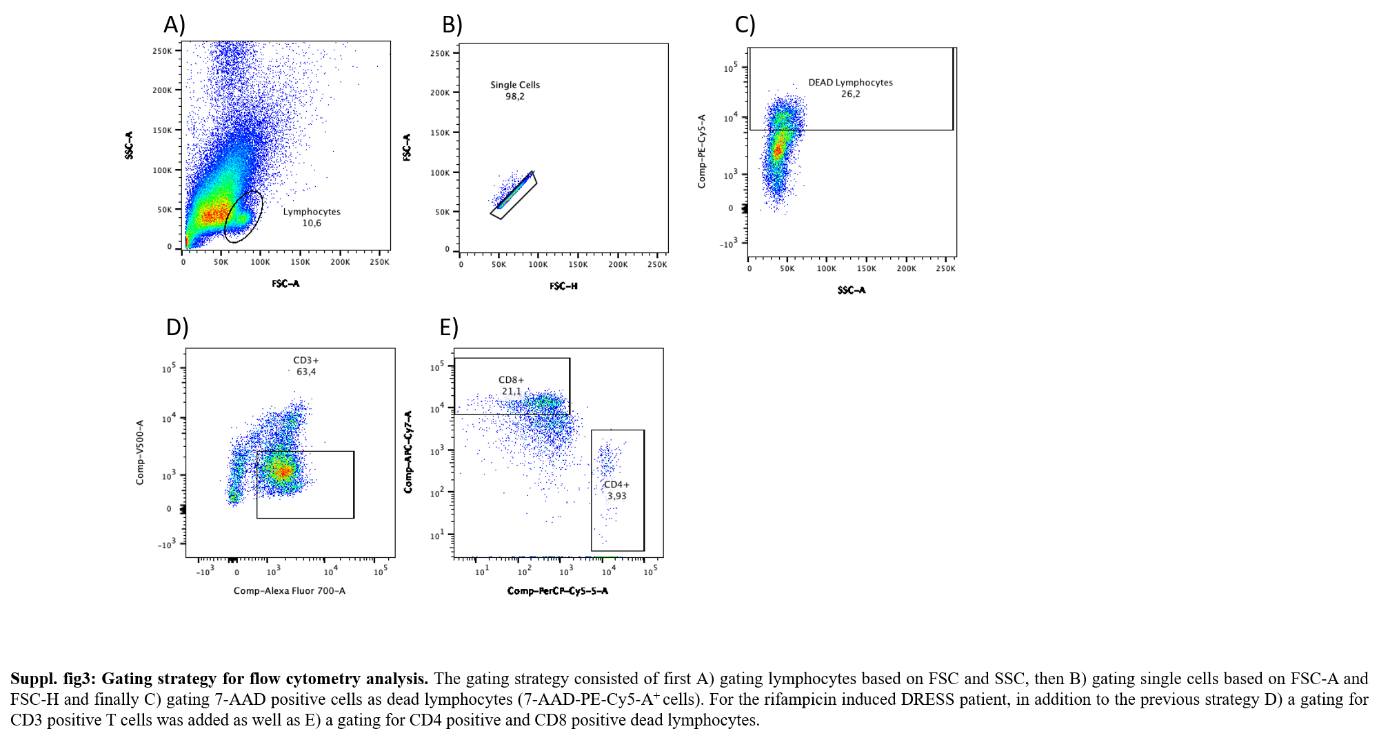

Supplement: Supplementary file 1 [file DataSheet1.docx]
